# When Helping Hurts: Nonabusing Family, Friends, and Neighbors in the Lives of Elder Mistreatment Victims

**DOI:** 10.1093/geroni/igaa057.2145

**Published:** 2020-12-16

**Authors:** Risa Breckman, David Burnes, Sarah Ross, Philip Marshall, J Jill Suitor, Mark Lachs, Karl Pillemer

**Affiliations:** 1 Weill Cornell Medicine/ NYC Elder Abuse Center, New York, New York, United States; 2 University of Toronto, Toronto, Ontario, Canada; 3 Cornell University, Ithaca, New York, United States; 4 Historic Preservation Program, Roger Williams University, Bristol, Rhode Island, United States; 5 Purdue University, West Lafayette, Indiana, United States; 6 Weill Cornell Medicine, New York City, New York, United States

## Abstract

Research conducted by the NYC Elder Abuse Center (NYCEAC) at Weill Cornell Medicine and colleagues found that concerned persons experience significant distress knowing about elder abuse and trying to assist victims. Data will be presented from a nationally representative survey which included items on concerned persons in elder abuse. Thirty-one percent of all respondents reported that they had a relative or friend who experienced elder abuse; of these, 61% had attempted to help the victim and over 80% reported the experience is very or extremely stressful (2017). By both knowing about and becoming involved in elder abuse situations, concerned persons experience significant emotional and practical problems and often need professional help. NYCEAC’s Elder Abuse Helpline for Concerned Persons is the first of its kind in the country. The Helpline’s services and structure will be explained, and possibilities for replication in other locations will be explored.

